# The Effect of Enzymatic Hydrolysis and Maillard Reaction on the Flavor of Chicken Osteopontin

**DOI:** 10.3390/foods13050702

**Published:** 2024-02-26

**Authors:** Xiong Xu, Ke Bi, Guangyu Wu, Ping Yang, Hongjun Li, Wei Jia, Chunhui Zhang

**Affiliations:** 1 College of Food Science, Southwest University, Chongqing 400715, China; x-xu@163.com (X.X.); lhj@swu.edu.cn (H.L.); 2 Key Laboratory of Agro-Products Processing, Ministry of Agriculture and Rural Affairs, Institute of Food Science and Technology, Chinese Academy of Agricultural Sciences, Beijing 100193, China; 82101211121@caas.cn (K.B.); wuguangyu1998@163.com (G.W.); yangping01@caas.cn (P.Y.); 18910106795@163.com (W.J.)

**Keywords:** chicken osteopontin, enzymatic hydrolysis, Maillard reaction, volatile compounds, non-volatile compounds

## Abstract

To reveal the changes in the flavor quality of chicken osteopontin (CO) before and after enzymatic hydrolysis and a thermal reaction, the present study was carried out to evaluate the volatile compounds and non-volatile compounds in CO. The results show that the chicken boneset enzymatic solution (CBES) presented a notably richer aroma after the enzymatic hydrolysis treatment. At the same time, the concentrations of the total free amino acids (FAAs) and 5′-nucleotides in the CBES increased dramatically. The ERP (enzymatic reaction paste) scores and the ORC (osteopontin reactive cream) scores were exceptionally high in terms of the umami and salty flavor profiles. As precursors, FAAs and 5′-nucleotides also boosted the Maillard reaction, leading to the generation of wide volatile compounds. Compared to CO, CBES, and ORC, the sensory evaluation showed that ERP scored the highest. In summary, the enzymatic hydrolysis treatment coupled with the Maillard reaction significantly enhanced the flavor profile of CO. These findings offer valuable insights into the high-value utilization of bone by-products, making a significant advancement in the field.

## 1. Introduction

In China, the world largest meat producer and consumer, the rapid development of the meat industry has brought about huge economic benefits [1]. However, this has been accompanied by the waste of a large number of bone by-products, the disposal of which has always been a problem [2]. For a long time, most animal bones were regarded as low-value by-products, and very often they can only be used as raw materials for feed or fertilizer. But, in fact, animal bone is a valuable biological resource, especially chicken bone, which is rich in protein, minerals, and other nutrients [3].

Bone residue is a primary bone extract produced from the processing of livestock and poultry bones and contains a large amount of protein, which can be used as a base to produce meat flavoring and can also be used in the processing industry to simulate the preparation of meat-flavoring products [4]. Chicken bone is a major by-product of poultry slaughter [5] and contains about 51% of moisture, 19% of protein (mainly collagen), and 15% of ash. Therefore, chicken bone has become a very valuable protein resource [6]. Chicken osteopontin (CO) is a seasoning product with a complex flavor obtained by processing chicken bones. Flavor modification is often required to obtain a meaty flavoring with a rich, rounded aroma and a mellow, realistic taste. Therefore, a derivatization treatment is required to enhance flavor quality. Enzymatic hydrolysis and Maillard reactions, as important units in the derivatization of osteopontin, play an important role in the regulation of osteopontin’s flavor [7]. In recent years, most studies have focused on the use of enzymatic hydrolysis to improve osteopontin utilization, aroma studies, and taste studies as well as on the use of a thermo-reactive treatment of osteopontin to enhance its flavor [8,9,10,11]. Ref. [4] demonstrated that hot-press extraction gave a higher protein recovery and a better product quality than the products obtained by other methods. The products subjected to bi-enzymatic sequential hydrolysis had higher hydrolysis degrees and yielded a higher content of small peptides and FAAs and, at the same time, reduced bitterness values [11]. It is reported that bone can be made more valuable by enzymatic hydrolysis, and more FAAs can be obtained by the enzymatic hydrolysis of bones [4]. The proteins, peptides, and free amino acids (FAAs) produced after enzymatic hydrolysis can provide rich flavor substances from chicken bones. At the same time, they provide flavor precursors for the Maillard reaction to produce a pleasant aroma. AMP, GMP, and IMP are the major flavor nucleotides in meat foods and contribute to the umami taste of meat [12]. It has been reported that alkaline protease combined with the flavor protease treatment of compound osteolysis hydrolysate increases their total nucleotide content and yields samples with a better flavor presentation [13].

In this paper, we used an electronic nose (E-nose) device and solid-phase microextraction combined with gas chromatography-mass spectrometry (GC-MS) to explore the effects of the types and concentrations of volatile components under different processing methods. An electronic tongue (E-tongue), FAAs, 5′-nucleotides, and taste activity values (TAVs) were used to evaluate the changes in non-volatile compounds. Ultimately, the changes in the flavor quality of CO before and after enzymatic hydrolysis and a thermal reaction were revealed to guide the preparation of natural meat flavor enhancers using a bone by-product of the industry.

## 2. Material and Methods

### 2.1. Chemicals

The standards of FAAs, 5′-nucleotides, methanol, potassium dihydrogen phosphate, and 2-methyl-3-heptanone were purchased from Sigma-Aldrich (Sigma-Aldrich, St. Louis, MO, USA). The reagents used in this paper were >99% pure. All the other chemicals used were of analytical grade, purchased from Sinopharm Chemical Reagent Beijing Co. Ltd., (Beijing, China).

### 2.2. Sample Preparation

**Figure 1:**
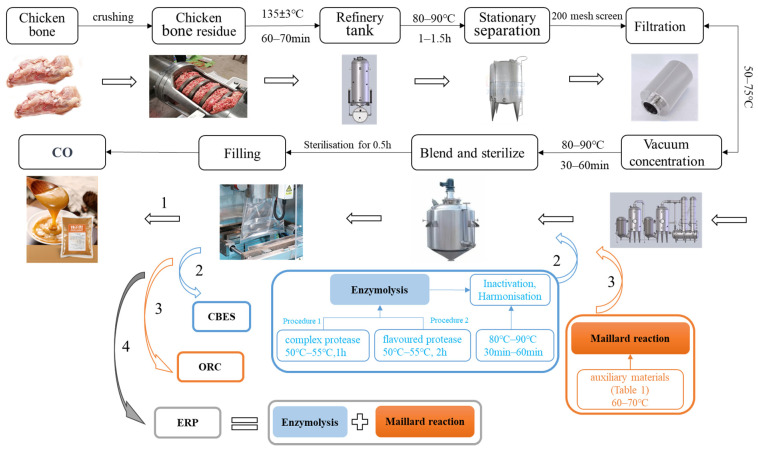
Flowchart of the preparation of CO (chicken osteopontin), CBES (chicken boneset enzymatic solution), osteopontin reactive cream (ORC), and enzymatic reaction paste (ERP).

All the samples were made in Henan Pulatay Biotechnology Co. (Hebi, China). The specific process parameters are shown in Figure 1 and Table 1.

CO: Put the chicken bone residue into the crusher (Shuncheng Fittings Co., Luoyang, China) for crushing, add water to the appropriate level, and heat up the refining tank (Pharmaceutical Machinery Factory Co., Changshu, China). After the constant temperature is reached, let it stand. The separated soup liquid will be extracted according to the sensory results. After all the extracted and separated soup liquid is filtered through a 200-mesh filter, it will be pumped into the vacuum-concentrated tank (Nanjing Canaan Pharmaceutical Co., Nanjing, China) for concentration; then, the concentration of the semi-finished products is sterilized (80–90 °C, 30–60 min) and the containers are filled.

CBES: On the basis of preparing CO vacuum concentration is carried out, and an enzyme is added for enzymatic hydrolysis (pH 6.8 ± 0.2). After enzymatic hydrolysis is finished, the temperature is raised to inactivate the enzyme and blend it evenly, and the product is sterilized and then filled the containers.

ORC: The CO is heated for the second time to further promote the Maillard reaction, the product is then sterilized, and filled the containers.

ERP: CBES is heated for the second time to further promote the Maillard reaction, the product is sterilized, and then filled the containers.

### 2.3. E-Tongue Analysis

Taste perception was analyzed using an Asrree II/LS16 E-tongue assay system (Alpha MOS Inc., Toulouse, France), a cross-sensitive potentiometric sensor array consisting of seven taste sensors—AHS (sour), CTS (savory), ANS (sweet), NMS (umami), SCS (bitter), PKS (general), and CPS (general). A total of 1 mL of sample was aspirated into 10 mL of water, centrifuged at 10,000 r/min for 15 min at 4 °C, and passed through a 0.45 μm filter membrane with a collection cycle of 1 time/s and a stirring rate of 3 r/s. Data were collected at room temperature.

### 2.4. FAAs Determination

The determination of the FAAs in this work referred to the method of [14], with some modifications. About 5 g of sample was weighed; 20 mL of deionized water was added and homogenized three times at 18,000 r/min in an ice bath (10 s each time, at 10 s intervals); 20 mL of 5% (*v*/*v*) a trichloroacetic acid (TCA) aqueous solution was added, mixed well, and left at 4 °C for 12 h. The filter was made on qualitative filter paper, and the filtrate was first adjusted to a pH of 6.0 with 4 mol/L KOH and then filtered through a filter membrane with 0.45 μm. We pipetted 10 μL of the filtrate obtained from the sample, added 70 μL of AccQ-Fluor buffer to the derivatization tube, then aspirated 20 μL of the ready-made AccQ-Fluor derivatization agent, added it to the derivatization tube, kept vortexing and mixing for 10 s, left it at room temperature for 1 min, heated it up in the oven at 55 °C for 10 min, then removed it to be injected into the sample, and, finally, determined the content of FAAs in the sample by RP-HPLC.

### 2.5. 5′-Nucleotides Determination

The determination of 5′-nucleotides in our study refers to the method of [15], with some modifications. Take 1 mL of sample in a 50 mL centrifuge tube, add 25 mL of ultrapure water, centrifuge it at 10,000 r/min, at 4 °C for 20 min, take the supernatant in a 50 mL centrifuge tube, fix the volume with ultrapure water, and pass it through a 0.22 μm filter membrane. Weigh 20 mg of 5′-AMP, 5′-GMP, and 5′-IMP standards, dissolve them in ultrapure water, and set them in 10 mL volumetric flasks to make standard solutions at a concentration of 2.0 mg/mL. Place 2.5, 5, 10, 20, 40, 80, 160, 320, 640, and 1280 μL of each standard solution in a 2 mL volumetric flask, fix the solutions with ultrapure water to 2 mL, shake well, and then filter through a 0.22 μm filter membrane. The HPLC conditions are as follows: HPLC column—Intersil ODS-3 (250 mm × 4.6 mm); column temperature—30 °C; UV detection wavelength—260 nm; and injection volume—10 μL. The mobile phase A is methanol, and B is 0.02 mol/L of a potassium dihydrogen phosphate solution (pH = 3.8). The mobile phase elution procedure is as follows: 5% A, 95% B, elution for 15 min, and a flow rate of 1 mL/min.

### 2.6. TAV Determination

The taste activity value (TAV) was calculated using the following equation: TAV = C/T.

In the above equation, C is the concentration of the umami taste substance, in mg/100 g, and T is the umami threshold of the umami taste substance, in mg/100 g (5′-IMP = 25 mg/100 g, 5′-AMP = 50 mg/100 g). Substances with a TAV exceeding 1 are identified as significant contributors to the flavor profile [16].

### 2.7. E-Nose Determination

The odor characteristics of CO were analyzed under different conditions using a PEN 3.5 E-nose system (AIRSENSE, Schwerin, Germany). A 10 mL sample was placed in a 20 mL headspace vial with an air inlet of 600 mL/min and detected by headspace sampling at room temperature, with a sensor cleaning time of 180 s and a measurement time of 60 s. The response values of the E-nose were obtained and analyzed using principal component analysis (PCA).

### 2.8. Determination of Volatile Compounds

The determination of the volatile compounds was based on the method of [17], with some modifications. The GC-MS analysis was conducted using a Thermo GC/MS setup, which included a TRIPLUS autosampler, a TRACE GC ULTRA gas chromatograph, and a DSQ mass-selective detector (Thermo Scientific, Waltham, MA, USA).

For this experiment, around 5 g of sample along with 2 mL of 1.11 mg/mL 2-methyl-3-heptanone were introduced into a 20 mL vial, which was then securely sealed. To extract the volatile compounds, a 75 mm HS-SPME fiber (Supelco, Bellefonte, PA, USA) was employed at a temperature setting of 50 °C for a duration of 30 min. Post collection, this fiber was placed into the injection port of the GC and subjected to a desorption process for 3 min at a temperature of 250 °C. The separation phase involved using a TR-5 MS capillary column (30 m length × 0.25 mm internaldia × 0.25 mm film thickness; Thermo Scientific, Waltham, MA, USA). The temperature inside the oven of the chromatograph was kept at 40 °C for the initial 3 min, then increased steadily by 3 °C/min up to 70 °C, followed by an increment of 5 °C/min until reaching 180 °C, and then a final ramp up of 10 °C/min up to 280 °C, where it remained for another 5 min. Helium served as the carrier gas, maintained at a constant flow of 1 mL/min. The energy for ionization was set to 70 electron volts, scanning a range of 50 to 550 *m*/*z*, and the ion source temperature was held at 230 °C. The retention time for each compound was converted to the LRI (linear retention index) using n-alkanes (C7~C30, purity ≥ 99.7%).

Identification of the compounds was achieved by matching their mass spectral profiles against the NIST 08 database, maintained by the National Institute of Standards and Technology in Gaithersburg, MD, USA, and by evaluating their LRI. The quantification of each volatile compound was carried out by contrasting the area of its peak in the chromatogram with that of the 2-methyl-3-heptanone internal standard.

The odor activity value (OAV) is calculated by comparing the concentration of a volatile compound to its threshold value in an aqueous solution. If a compound has an OAV exceeding 1, it is recognized as playing a key role in contributing to the overall aroma.

### 2.9. Sensory Evaluation

Sensory evaluation was executed following the previous method [18]. Sensory evaluation was performed by 12 trained panelists recruited from the Institute of Food Science and Technology, Chinese Academy of Agricultural Sciences (Beijing, China). A week-long training was conducted to ensure that the panelists could accurately identify the aroma of the samples. After discussion, the panelists described the flavor attributes, including caramel, grassy, greasy, roasted, and meaty flavors. The specific description and training standards of each attribute were as follows: 2,5-dimethyl-4-hydroxy-3(2H)-furanone (caramel), grass camphor (grassy), 2,4-Heptadienal (greasy), 2-methylpyrazine (roasted), and the smell of boiled chicken breast (meaty). Each attribute was evaluated on a 0−3 point scale with steps of 0.5. Necessary rest (1 min) was required between samples to restore sensation. Coffee beans were prepared to restore the panelists’ sense of smell. The aroma evaluators were blinded to the nature of the samples tested, and each sample was evaluated three times by each panel member. The final score was expressed as the mean of three replicate scores.

### 2.10. Statistical Analysis

The IBM SPSS Statistics 26.0 software was used for data analysis. The Origin 8.0 software was used for graphing. Our correlation analysis used the R 4.0.1 statistical 250 software (R Foundation for Statistical Computing, Sydney, Austria) to detect the relationship between the samples and the experimental data. The index measurements were all parallel results repeated three times. The differences were significant at *p* < 0.05.

## 3. Results

### 3.1. Non-Volatile Compounds Analysis

#### 3.1.1. E-Tongue

**Figure 2:**
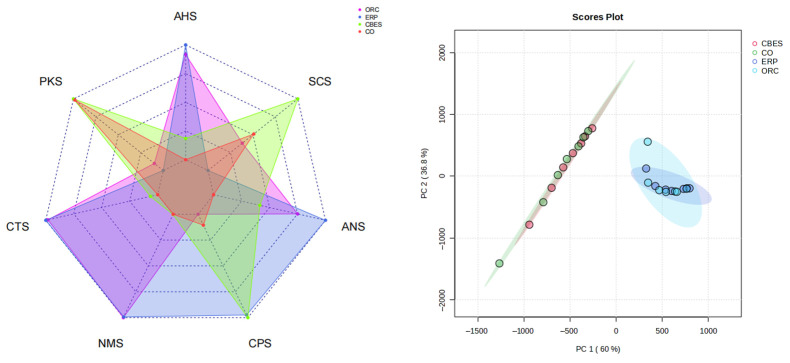
Radar plot and principal component analysis of the electronic-tongue (E-tongue) data on chicken osteopontin under different treatment conditions. AHS (sour), CTS (salty), ANS (sweet), NMS (umami), SCS (bitter), PKS (general), and CPS (general). CO (chicken osteopontin), CBES (chicken boneset enzymatic solution), osteopontin reactive cream (ORC), and enzymatic reaction paste (ERP).

Figure 2 shows the taste response intensity of CO to sour, salty, sweet, umami, and bitter tastes under different treatment conditions. It can be seen from the figure that there are certain differences in the response intensity of the samples to different sensors. Among them, CBES is the most sensitive to the bitter taste sensor, while ORC and ERP are more responsive to the sour, umami, and sweet taste sensors, indicating that these three tastes are the main tastes of ORC and ERP. The intensity of ORC on the sweetness sensor is lower than that of ERP, which may be due to the fact that the enzymatically treated ORC can change the abundance of taste substances in the Maillard reaction’s product.

The PCA diagram of the E-tongue shows that PC1 and PC2 contribute 60% and 36.8%, respectively, with a cumulative contribution of 96.8% (Figure 2). Therefore, PC1 and PC2 can reflect the taste characteristics of the sample. It can be seen from the figure that the contribution rate of PC1 is greater than that of PC2, indicating that the taste difference of different samples is mainly determined by the first principal component. There is an overlap between ORC and ERP, as there is overlap between CO and CBES, indicating that there is a certain similarity in their taste components.

#### 3.1.2. FAAs Analysis

FAAs are important active substances in organisms and the basic units of enzymes and proteins. Volatile compounds are produced through the Maillard reaction to improve the taste and aroma of meat products. At the same time, Maillard reaction can increase the content of umami-related amino acids [19]. The production of amino acids from protein degradation is the main cause of flavor formation [20] and is also an important precursor of aromatic compounds [21].

This study compared seventeen FAAs in CO under four different treatment conditions (Table 2). Among the 17 FAAs, Asp and Glu were umami; Ser, Pro, Gly, Thr, and Ala were sweet; and Val, Met, Ile, Phe, Lys, Leu, Arg, His, and Tyr were all bitter. Cys is an odorless amino acid [22]. Although some taste substances had a low concentration in the test sample, they had a great impact on the taste due to their low threshold. To evaluate the impact of these compounds on taste intensity, TAV is a very useful metric (Table 3). The FAAs with higher concentrations in ORC and ERP included Ser, Gly, Glu, Ala, and Cys, but the main FAAs were Ala and Glu (TAV > 1). It was suggested that these two FAAs may be important components of ORC and ERP. This is consistent with previously detected E-tongue data. As it can be seen from the table, the total content of sweet amino acids and umami amino acids in CBES was significantly higher than that of CO, while the total amount of bitter amino acids was lower than that of CO, which showed that the taste of CO was further improved after enzymatic hydrolysis. This is consistent with the research results of [9]. By comparing the total amino acid content, it can be found that the enzymatic hydrolysis treatment can increase the content of FAAs in CO. Compared with CO and CBES, ORC and ERP have a stronger umami and sweet taste. It has been reported that, when alanine and glycine come into contact with sweet substance receptors, we can feel a stronger sweetness, and they can offset salty and bitter tastes [23]. White sugar is added when preparing ORC and ERP, which may be one of the main reasons why ORC and ERP have a more obvious sweetness than CO and CBES.

#### 3.1.3. 5′-Nucleotide Analysis

The concentrations of the 5′-nucleotides (5′-AMP, 5′-IMP, 5′-GMP, and 5′-CMP) are shown in Table 2. Ref. [24] reported that 5′-AMP, 5′-CMP, 5′-IMP, and 5′-GMP can impart a rich flavor to foods. At the same time, they can synergize with MSG to form a mellow taste and a stronger umami taste [25]. MSG was added during the preparation of ORC and ERP, which made the taste of the samples more intense. 5′-GMP and 5′-IMP help enhance the umami flavor, while 5′-AMP enhances sweetness. AMP and IMP have significant effects on the taste of ORC and ERP (TAV > 1). GMP has a small contribution to the umami taste of CO (TAV < 1). ERP has the highest 5′-AMP content.- there may be some cellular material in bone residue, and enzymatic treatment destroys its cell membrane, causing more nucleotides to dissolve. On the other hand, ATP thermally decomposes during boiling to form a large amount of AMP, which imparts a strong sweet taste [26].

### 3.2. Volatile Compounds Analysis

#### 3.2.1. E-Nose

**Figure 3:**
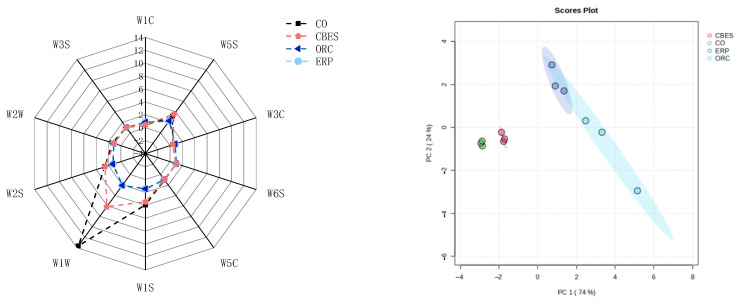
Radar plot and principal component analysis of the electronic-nose (E-nose) data on chicken osteopontin under different treatment conditions. W1C is sensitive to aromatic compounds. W5S is sensitive to nitrogen oxide compounds. W3C is sensitive to ammonia and aromatic compounds. W6S is sensitive to hydrides. W5C is sensitive to short-chain alkanes and aromatic compounds. W1S is sensitive to methyl compounds. W1W is sensitive to alcohols, aldehydes, and ketones. W2S is sensitive to inorganic sulfides. W2W is sensitive to aromatic components and organic sulfides. W3S is sensitive to long-chain alkanes. CO (chicken osteopontin), CBES (chicken boneset enzymatic solution), osteopontin reactive cream (ORC), and enzymatic reaction paste (ERP).

Figure 3 shows the E-nose response diagram of CO under different treatment conditions. As it can be seen from the figure, the aroma profiles of CO and CBES are similar, and the aroma profiles of ORC and ERP are similar. Among the 10 sensors of the E-nose, W1W has a higher response value and has obvious changes after enzymatic hydrolysis and the heat treatment, indicating that it is more sensitive to changes in alcohols, aldehydes, and ketones.

The PCA of the E-nose signal data of volatile flavor substances in the CO under different treatment conditions was carried out, and the differentiation results are shown in Figure 3. The sum of the PC1 (74%) and the PC2 (contribution rate 24%) (contribution rate 98%) represents that the information of the sample collected by the E-nose can reflect almost all of its volatile information. The overlapping measurements of ORC and ERP indicate that there is a certain similarity in their flavor compounds. Therefore, GC-MS was run to further identify the effects on the volatile flavor compounds of CO before and after the Maillard reaction and enzymatic hydrolysis.

#### 3.2.2. Volatile Compounds

As it can be seen from Table 4, forty-five volatile compounds with nine categories were detected in the CO, including aldehydes, alcohols, ketones, pyrazines, furans, pyrrolyl, thiazoles, sulfurs, acids, esters, and other compounds. At the same time, aldehydes, alcohols, furans, esters, acids, and other compounds showed a significant increase after the Maillard reaction. There was no significant change in pyrrolyl and thiazoles.

The amount of alcohols and aldehydes was the highest among the various aroma compounds. From Table 5, a total of 13 aroma compounds with OAVs greater than 1 were found, containing octanal, nonanal, 1-octen-3-ol, linalool, estragole, 2-phenylethanol, 3,6-dimethyl-2-ethylpyrazine, 2-acetylthiazole, 2-methyl-3-furanthiol, 3,3′-dithiobis-2-methylfuran, 2-methyl-naphthalene, 2-methoxy-phenol, and *p*-cresol. Among the sulfur-containing compounds, especially 2-methyl-3-furanthiol and 3,3′-dithiobis-2-methyl-furan had high OAVs of 5004 and 214,942, respectively, which may be related to the degradation of sulfur-containing FAAs in CO through the Maillard reaction.

Despite the abundance of esters and other compounds in the samples, the compounds were detected at low concentrations and high odor thresholds, so they contributed less to the perception of CO aroma. Therefore, these compounds were not analyzed in detail in this paper.

### 3.3. Relationships among the Variables of Taste and Aroma Traits

**Figure 4:**
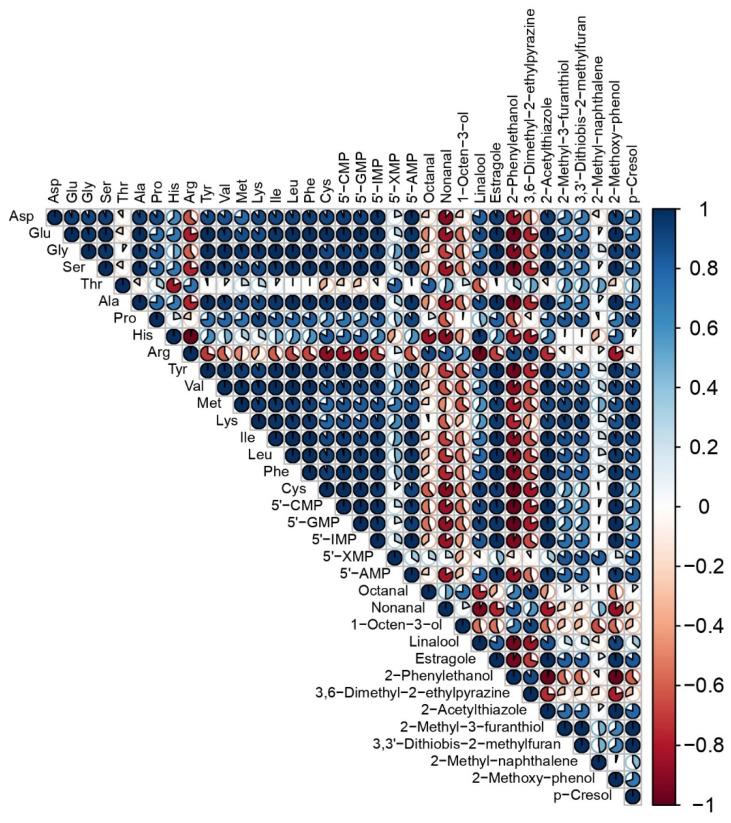
Correlation analysis of the samples and experimental data. The positive and negative correlation coefficients (r) are colored in blue and red, respectively. The fraction of the circles stands for the correlation coefficient, and a full fraction means r = 1.

A correlation analysis was used to evaluate the relationship between taste-related substances (FAAs, 5′-nucleotides) and key volatile compounds (OAV > 1) (Figure 4). Regarding the correlation of taste substances, we found that there was a positive correlation between the 17 FAAs and the 5′-nucleotides, except for arginine. Regarding the correlation between taste and aroma, it could be observed that, among the thirteen key volatile compounds, five compounds were negatively correlated. This showed that, except for arginine, the other FAAs and the 5′-nucleotides promoted the Maillard reaction and the generation of flavor precursors under the action of enzymatic hydrolysis.

## 4. Discussion

### 4.1. Taste Changes in CO under Different Treatment Conditions

FAAs and 5′-nucleotides play unique and important roles in the flavor formation of meat extracts such as chicken bone extracts. This article studied the taste changes in CO after enzymatic hydrolysis and heat treatment by measuring the above indicators [8]. CBE adds complex protease and flavor protease on the preparing of CO. Complex protease is a mixture of multiple proteases that can work under different pH and temperature conditions to hydrolyze proteins more comprehensively. By destroying the protein structure and improving its water solubility, food has a better taste during processing and consumption [28]. Flavor proteases are mainly used for flavor enhancement. This type of protease is specialized in producing specific flavor compounds. They precisely cleave proteins and release FAAs and small peptides with flavor-enhancing properties. These components interact with each other in different ways in chicken bone residue, which, together, constitute its complex taste characteristics [29]. By precisely controlling the proportions and processing conditions of these ingredients, the final flavor of CO can be adjusted to create products that meet specific taste needs. In food processing and formulation design, the scientific understanding and application of these ingredients is the key to optimizing the taste of food. At the same time, FAAs with specific flavors, such as glutamic acid, can be released to enhance the umami taste of food [30]. The application of these two types of enzymes in food processing can makethem complement each other. Through precise formulas and processing conditions, the flavor and texture of food can be greatly improved and regulated [31]. In addition, the Maillard reaction can be promoted by adding white sugar, glucose, cysteine, alanine, and other Maillard reaction precursors to the preparing of CO, ultimately producing rich flavor components. ERP is based on CBE and further enhances the flavor by strengthening enzymatically hydrolyzed CO.

**Figure 5:**
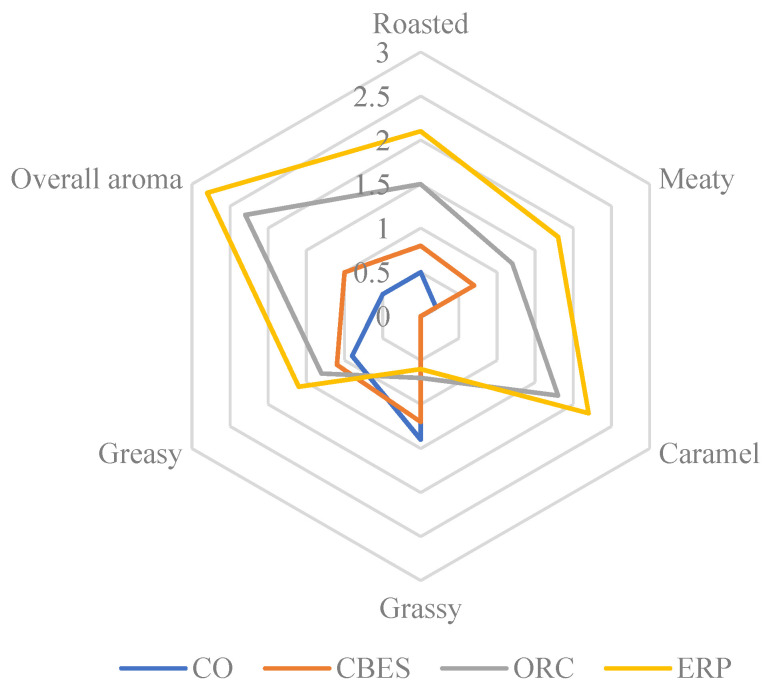
The aroma profiles analyzed by the sensory evaluation of chicken osteopontin under different treatments. CO (chicken osteopontin), CBES (chicken boneset enzymatic solution), osteopontin reactive cream (ORC), and enzymatic reaction paste (ERP).

After sensory evaluation (Figure 5), it was found that ERP has a more mellow flavor and have a higher acceptance rate among the panelists. The aroma is more intense, with typical roasting and meat aroma characteristics. Bones are a good source of minerals and are rich in FAAs. From Table 2, we can find that the FAAs content of ERP is significantly higher than that of CO, CBES, and ORC, which may be related to the continued heating and pressure (Maillard-enhanced reaction) in the later stage of processing and the addition of yeast [32]. The metabolic activity of yeast can release more FAAs and nucleotides, which are important components and enhance the umami taste of food, especially the Ala and Glu contents, reach values of 4.08 and 5.44, respectively. Ala is the key amino acid that increases umami taste. It is the main component of natural MSG and can provide an obvious umami taste. Glu usually imparts sweetness to meat and plays a balancing role in interactions with other flavor components [33]. 5′-Nucleotides play a vital role in enhancing the flavor of meat extracts. These compounds, especially 5′-IMP and 5′-AMP, are widely used as flavor enhancers in the food industry. In ERP, the TAVs of 5′-IMP and 5′-AMP reached 1.39 and 1.35, respectively, which shows that 5′-IMP and 5′-AMP are key taste-producing substances and provide the umami taste of CO. In addition, 5′-IMP and 5′-AMP work synergistically with glutamic acid to significantly enhance the umami taste of food. 5′-IMP, 5′-AMP, and glutamate work together to produce an umami taste that is much greater than the sum of their separate effects [34].

### 4.2. Aroma Changes in CO under Different Treatment Conditions

According to Table 4, we can find that the total content of aldehydes and alcohols increased to varying degrees after different treatments, mainly due to the addition of a large number of flavor-producing precursors to ORC and ERP, including Ala, glucose, starch, and yeast. Among them, glucose can directly participate in the Maillard reaction. Starch can be hydrolyzed into glucose monosaccharides to participate in the Maillard reaction. The addition of yeast can release more FAAs and nucleotides, causing the Maillard reaction to continue and produce a large number of aldehydes and alcohols [33]. It is worth noting that the amount of octanal and nonanal decreased, which may be due to lipid oxidation, leading to a decrease in unsaturated fatty acid content. Moreover, the addition of MSG and yeast may inhibit the oxidation of fatty acids [35]. Linalool and estragole are not produced through the biochemical processes of meat, but from the seasoning and cooking processes of meat. These two alcohols are often used as components of spice complexes. After being added, they can increase the harmony of the aroma of CO and enhance the flavor [36]. The contents of 2-phenylethanol in ORC and ERP are significantly reduced. As the Maillard reaction proceeds, they can compete with other compounds and be converted into different compounds. During high-temperature cooking or processing, these compounds may undergo thermal degradation, resulting in a reduction in their content [37].

Sulfur-containing compounds have a significant impact on the odor of CO, and, due to their extremely low threshold, they can impart complexity and depth to the meaty flavor of a product [38]. The significant increase in the concentrations of 2-methyl-3-furanthiol and 3,3′-dithiobis-2-methyl-furan among the sulfur-containing compounds is mainly due to the addition of flavor protease and complex protease, which degrade FAAs and produce rich sulfur amino acids, i.e., cysteine and methionine. They can further react with reducing sugars in the Maillard reaction to produce sulfur-containing compounds with specific flavors and are further hydrolyzed to produce sulfur-containing compounds during heating and enzyme catalysis [39]. The further degradation of unsaturated fatty acids may also be one of the main reasons for the oxidation of fat in CO, producing sulfur-containing compounds.

With the further intensification of the Maillard reaction, pyrazine gradually undergoes deamination reactions, cyclization reactions, polymerization, and redox reactions in the later stages of the reaction, further producing more complex nitrogen-containing heterocyclic compounds such as thiazole and pyridine [40].

## 5. Conclusions

The enzymatic hydrolysis treatment of CO markedly enhances the Maillard reaction. This process significantly raises the FAAs and 5′-nucleotides levels in CO, supplying abundant precursors for the Maillard reaction. Such treatment not only refines the fundamental flavor profile of the CO but also, by intensifying the Maillard reaction, further escalates the concentration of volatile compounds like octanal and nonanal. This advancement is vital for improving the flavor and value of bone by-products, offering a novel approach to flavor enhancement in the food industry. In the future, optimal conditions for enzymatic hydrolysis and the Maillard reaction could be investigated to enhance the flavor of CO as well as explore the application of CO in various food products.

## Figures and Tables

**Table 1:** Formulation table for samples.

Auxiliary Materials	Weights (kg/t)			
	CO	CBES	ORC	ERP
CO	—	1000	700	700
Complex protease	—	0.23	—	0.23
Flavor protease	—	0.38	—	0.38
Chicken oil	—	—	150	150
Yeast extract	—	—	80	80
White Sugar	—	—	10	10
Glucose	—	—	10	10
Monosodium glutamate	—	—	10	10
Cysteine	—	—	5	5
VB_1_	—	—	5	5
Alanine	—	—	5	5
Starch	—	—	15	15
Salt	—	—	10	10

Note: “—” indicates not added. CO (chicken osteopontin), CBES (chicken boneset enzymatic solution), osteopontin reactive cream (ORC), and enzymatic reaction paste (ERP).

**Table 2:** The taste compounds of chicken osteopontin under different treatment conditions.

Taste Compounds	Concentrations (mg/mL)			
	CO	CBES	ORC	ERP
**Amino acid**				
**Umami amino acids**				
Asp	67.67 ± 0.68 ^b^	95.45 ± 42.73 ^ab^	124.45 ± 19.13 ^a^	140.69 ± 14.77 ^a^
Glu	139.05 ± 5.94 ^c^	152.35 ± 29.72 ^c^	1317.57 ± 241.9 ^b^	1632.07 ± 132.86 ^a^
**Subtotal**	206.72 ± 6.62 ^d^	247.8 ± 72.45 ^c^	1442.02 ± 261.03 ^b^	1772.76 ± 147.63 ^a^
**Sweet amino acids**				
Gly	255.7 ± 44.63 ^b^	253.17 ± 47.27 ^b^	354.39 ± 99.69 ^ab^	454.67 ± 68.16 ^a^
Ser	141.84 ± 8.92 ^b^	152.15 ± 31.11 ^b^	642.69 ± 117.13 ^a^	797.56 ± 121.14 ^a^
Thr	333.94 ± 64.39	337.12 ± 57.43	261.6 ± 74.81	358.87 ± 48.57
Ala	191.9 ± 16.68 ^c^	203.58 ± 43.28 ^c^	1879.95 ± 421.88 ^b^	2447.27 ± 364.01 ^a^
**Subtotal**	994.8 ± 144.73 ^d^	1022.66 ± 193.31 ^c^	3214.58 ± 735.47 ^b^	4140.46 ± 618.19 ^a^
**Bitter amino acids**				
Pro	71.42 ± 10.11	76.64 ± 14.22	75.95 ± 21.96	82.09 ± 16.31
His	31.46 ± 4.97 ^c^	32.57 ± 6.65 ^c^	142.88 ± 27.7 ^b^	70.15 ± 12.85 ^a^
Arg	414.78 ± 88.7	439.51 ± 70.75	314.57 ± 88.84	375.36 ± 39.00
Tyr	54.16 ± 15.49	44.9 ± 5.18	92.85 ± 23.61	117.99 ± 16.97
Val	56.91 ± 8.42 ^b^	35.62 ± 19.51 ^b^	125.66 ± 32.29 ^a^	212.71 ± 33.11 ^a^
Met	19.54 ± 3.26 ^bc^	9.01 ± 1.00 ^c^	27.56 ± 13.21 ^b^	53.47 ± 11.54 ^a^
Lys	55.31 ± 3.45 ^b^	61.68 ± 13.5 ^b^	73.32 ± 13.41 ^b^	106.18 ± 15.22 ^a^
Ile	35.98 ± 3.63 ^c^	33.44 ± 4.86 ^c^	93.84 ± 21.33 ^b^	154.27 ± 24.28 ^a^
Leu	70.9 ± 9 ^c^	60.37 ± 10.83 ^c^	279.64 ± 71.08 ^b^	433.46 ± 72.75 ^a^
Phe	35.63 ± 8.13 ^c^	38.65 ± 4.67 ^c^	111.7 ± 29.35 ^b^	162.38 ± 24.6 ^a^
**Subtotal**	774.67 ± 145.05 ^c^	755.75 ± 136.95 ^d^	1262.02 ± 320.82 ^b^	1685.97 ± 250.32 ^a^
**Tasteless amino acids**				
Cys	12.39 ± 2.24 ^b^	12.77 ± 2.34 ^b^	775.49 ± 328.24 ^a^	756.96 ± 112.41 ^a^
**Total**	1988.58 ± 298.64 ^d^	2038.98 ± 405.05 ^c^	6694.11 ± 1626.43 ^b^	8356.15 ± 1128.55 ^a^
**Nucleotide**				
5′-CMP	0.22 ± 0.01 ^c^	0.28 ± 0.04 ^c^	20.83 ± 1.07 ^b^	24.53 ± 1.17 ^a^
5′-GMP	—	—	0.91 ± 0.04 ^b^	1.00 ± 0.04 ^a^
5′-IMP	19.56 ± 1.08 ^d^	22.66 ± 1.07 ^c^	33.78 ± 1.67 ^b^	39.11 ± 2.21 ^a^
5′-XMP	62.43 ± 3.70 ^ab^	57.86 ± 0.62 ^bc^	55.54 ± 3.27 ^c^	67.53 ± 3.68 ^a^
5′-AMP	49.42 ± 2.47 ^d^	57.22 ± 1.08 ^c^	69.56 ± 3.56 ^b^	79.84 ± 3.81 ^a^
**Total**	131.63 ± 7.26 ^d^	138.02 ± 2.8 ^c^	180.62 ± 9.61 ^b^	212.01 ± 10.91 ^a^

Note: Each value is expressed as mean ± SD (*n* = 3). Means in the same row with no common superscript differ significantly (*p* < 0.05). CO (chicken osteopontin), CBES (chicken boneset enzymatic solution), osteopontin reactive cream (ORC), and enzymatic reaction paste (ERP). “—” indicates that the substance has not been detected. The absence of letters indicates no significant difference between groups.

**Table 3:** The taste threshold and TAV of the taste compounds in chicken osteopontin under different treatment conditions.

Taste Compounds	Taste Threshold in Water (mg/mL)	TAV			
		CO	CBES	ORC	ERP
**Amino acid**					
Asp	1	0.07	0.1	0.12	0.14
Ser	1.5	0.09	0.1	0.43	0.53
Glu	0.3	0.46	0.51	4.39	5.44
Gly	1.3	0.20	0.19	0.27	0.35
His	0.2	0.16	0.16	0.71	0.35
Arg	0.5	0.83	0.88	0.63	0.75
Thr	2.6	0.13	0.13	0.10	0.14
Ala	0.6	0.32	0.34	3.13	4.08
Pro	3.0	0.02	0.03	0.03	0.03
Cys	—	—	—	—	—
Tyr	—	—	—	—	—
Val	0.4	0.14	0.09	0.31	0.53
Met	0.3	0.07	0.03	0.09	0.18
Lys	0.5	0.11	0.12	0.15	0.21
Ile	0.9	0.04	0.04	0.10	0.17
Leu	1.9	0.04	0.03	0.15	0.23
Phe	0.9	0.04	0.04	0.12	0.18
**Nucleotide**					
5′-AMP	50	0.99	1.14	1.39	1.6
5′-GMP	12.5	—	—	0.07	0.08
5′-IMP	25	0.78	0.91	1.35	1.56

Note: Asp, aspartic acid; Thr, threonine; Ser, serine; Glu, glutamic acid; Gly, glycine; Ala, alanine; Cys, cysteine; Val, valine; Met, methionine; Ile, isoleucine; Leu, leucine; Tyr, tyrosine; Phe, phenylalanine; Lys, lysine; His, histidine; Arg, arginine; and Pro, proline. CO (chicken osteopontin), CBES (chicken boneset enzymatic solution), osteopontin reactive cream (ORC), and enzymatic reaction paste (ERP). “—” indicates that the substance has not been detected.

**Table 4:** Effect of different treatment conditions on the aroma components of chicken osteopontin.

No.	Compounds	LRI	Identification	Concentration (ng/g)			
				CO	CBE	ORC	ERP
***Aldehyde***							
1	Octanal	1287	MS + RI	1.19 ± 0.12 ^b^	2.44 ± 0.04 ^a^	—	1.54 ± 0.01 ^b^
2	Nonanal	1390	MS + RI	19.86 ± 0.29 ^a^	13.37 ± 0.63 ^b^	3.70 ± 0.32 ^d^	8.16 ± 0.70 ^c^
3	Furfural	1455	MS + RI	2.90 ± 0.20 ^b^	4.10 ± 0.44 ^a^	—	—
4	Benzaldehyde	1508	MS + RI	—	26.79 ± 0.34 ^c^	37.68 ± 1.24 ^b^	55.40 ± 2.40 ^a^
5	5-Methyl-thiophene-2-carboxaldehyde	1755	MS + RI	—	—	15.22 ± 0.67 ^b^	36.76 ± 1.30 ^a^
6	2-Carboxaldehyde pyrrole-	2059	MS + RI	—	—	1.05 ± 0.04	0.66 ± 0.03
	**Subtotal**			23.95 ± 0.61 ^d^	46.70 ± 1.45 ^c^	57.65 ± 2.27 ^b^	102.52 ± 4.44 ^a^
***Alcohols***							
1	1-Octen-3-ol	1442	MS + RI	—	1.15 ± 0.02	—	—
2	2-Ethyl-hexanol	1480	MS + RI	6.75 ± 1.05 ^a^	—	—	1.77 ± 0.33 ^b^
3	Linalool	1552	MS + RI	—	—	5.54 ± 0.20 ^a^	3.50 ± 0.30 ^b^
4	1-Octanol	1561	MS + RI	6.78 ± 1.03 ^a^	7.46 ± 1.24 ^a^	1.89 ± 0.08 ^c^	2.36 ± 0.26 ^c^
5	Diethylene Glycol Mono-ethyl-Ether	1619	MS + RI	—	—	4.92 ± 0.60 ^b^	7.47 ± 0.45 ^a^
6	2-Furanmethanol	1669	MS + RI	11.00 ± 1.35 ^b^	10.61 ± 1.00 ^b^	8.73 ± 0.87 ^c^	22.23 ± 2.03 ^a^
7	Estragole	1685	MS + RI	—	—	9.73 ± 1.02 ^b^	15.61 ± 1.25 ^a^
8	Alpha-Terpineol	1690	MS + RI	—	—	6.72 ± 0.60	5.92 ± 0.52
9	Benzyl alcohol	1878	MS + RI	1.36 ± 0.06 ^d^	2.30 ± 0.35 ^c^	16.01 ± 1.50 ^b^	19.59 ± 1.08 ^a^
10	Phenylethyl alcohol	1919	MS + RI	0.91 ± 0.05	1.14 ± 0.04	—	—
	**Subtotal**			26.79 ± 3.54 ^c^	22.65 ± 2.65 ^d^	53.54 ± 4.87 ^b^	78.45 ± 6.22 ^a^
***Ketones***							
1	Acetophenone	1645	MS + RI	7.00 ± 1.06 ^a^	1.72 ± 0.13 ^b^	—	—
2	N-Methyl-2-pyrrolidone	1662	MS + RI	—	3.17 ± 0.46 ^a^	1.95 ± 0.22 ^b^	3.18 ± 0.30 ^a^
	**Subtotal**			7.00 ± 1.06 ^a^	4.89 ± 0.59 ^b^	1.95 ± 0.22 ^d^	3.18 ± 0.30 ^c^
***Pyrazines***							
1	2-Methylpyrazine	1276	MS + RI	1.73 ± 0.07 ^b^	1.86 ± 0.03 ^b^	1.56 ± 0.04 ^c^	2.85 ± 0.12 ^a^
2	2,6-Dimethylpyrazine	1319	MS + RI	26.69 ± 1.26 ^a^	24.03 ± 1.22 ^a^	5.17 ± 0.78 ^c^	9.71 ± 1.20 ^b^
3	2,5-Dimethylyrazine	1323	MS + RI	—	—	5.43 ± 0.20	6.05 ± 0.32
4	2,3-Dimethylpyrazine	1346	MS + RI	—	—	1.28 ± 0.05	1.91 ± 0.03
5	6-Methyl-2-ethylpyrazine	1390	MS + RI	1.62 ± 0.05 ^c^	1.75 ± 0.07 ^c^	2.50 ± 0.20 ^b^	4.76 ± 0.43 ^a^
6	5-Methyl-2-ethylpyrazine	1399	MS + RI	2.89 ± 0.25 ^b^	3.87 ± 0.44 ^a^	1.6 ± 0.19 ^c^	2.86 ± 0.25 ^b^
7	Trimethylpyrazine	1413	MS + RI	6.86 ± 0.74 ^d^	7.48 ± 0.75 ^c^	11.43 ± 1.10 ^b^	15.08 ± 1.55 ^a^
8	2,5-Dimethyl-3-ethylpyrazine	1449	MS + RI	4.22 ± 0.42 ^b^	6.48 ± 0.65 ^a^	2.37 ± 0.20 ^c^	3.37 ± 0.34 ^b^
	**Subtotal**			44.01 ± 2.79 ^a^	45.47 ± 3.16 ^a^	31.41 ± 2.76 ^b^	46.6 ± 4.24 ^a^
***Furans***							
1	2-Pentylfuran	1249	MS + RI	—	1.60 ± 0.08 ^b^	1.02 ± 0.12 ^b^	5.60 ± 0.49 ^a^
2	2-Acetylfuran	1479	MS + RI	—	—	37.12 ± 2.33 ^b^	49.51 ± 1.5 ^a^
	**Subtotal**			—	1.60 ± 0.08 ^c^	38.14 ± 2.45 ^b^	55.11 ± 1.99 ^a^
***Pyrrole, thiazole***							
1	2-Acetylthiazole	1643	MS + RI	—	—	5.05 ± 0.39 ^b^	7.02 ± 0.89 ^a^
2	Benzothiazole	1961	MS + RI	3.45 ± 0.40 ^a^	1.62 ± 0.12 ^b^	0.98 ± 0.03 ^c^	0.80 ± 0.09 ^c^
3	2-Acetylpyrrole	1971	MS + RI	1.57 ± 0.12 ^b^	1.77 ± 0.20 ^b^	26.26 ± 2.04 ^a^	23.15 ± 1.98 ^a^
	**Subtotal**			5.02 ± 0.52 ^b^	3.39 ± 0.32 ^b^	27.24 ± 2.07 ^a^	23.95 ± 2.07 ^a^
***Sulfurs***							
1	Ethanethioic acid S-ethyl ester	1109	MS + RI	—	—	—	2.32 ± 0.28
2	2-Methyl-3-furanthiol	1315	MS + RI	—	—	—	2.00 ± 0.09
3	Bis(2-Methyl-3-furyl) disulfide	2100	MS + RI	—	—	—	4.30 ± 0.38
	**Subtotal**			—	—	—	8.62 ± 0.75
***Acids***							
1	Acetic acid	1460	MS + RI	1.96 ± 0.89 ^c^	1.85 ± 0.85 ^c^	18.58 ± 1.56 ^b^	49.72 ± 4.25 ^a^
2	Isovaleric acid	1680	MS + RI	—	—	5.98 ± 0.34 ^b^	8.3 ± 0.55 ^a^
3	Heptanoic acid	1934	MS + RI	—	—	2.09 ± 0.10	2.22 ± 0.13
4	Hexanoic acid	1954	MS + RI	—	—	6.45 ± 0.67 ^b^	8.40 ± 0.85 ^a^
	**Subtotal**			1.96 ± 0.89 ^c^	1.85 ± 0.85 ^c^	33.10 ± 2.67 ^b^	68.64 ± 5.78 ^a^
***Esters***							
1	Butyrolactone	1626	MS + RI	2.05 ± 0.31 ^c^	1.82 ± 0.23 ^c^	3.04 ± 0.35 ^b^	5.02 ± 0.67 ^a^
2	5-Decanolide	2220	MS + RI	0.53 ± 0.05 ^b^	0.70 ± 0.05 ^b^	1.26 ± 0.16 ^a^	1.90 ± 0.20 ^a^
	**Subtotal**			2.58 ± 0.36 ^c^	2.52 ± 0.28 ^c^	4.30 ± 0.51 ^b^	6.92 ± 0.87 ^a^
***Others***							
1	D-Limonene	1105	MS + RI	—	—	3.89 ± 0.42 ^b^	9.16 ± 0.92 ^a^
2	Styrene	1254	MS + RI	1.80 ± 0.20	1.79 ± 0.18	—	—
3	Naphthalene	1707	MS + RI	—	—	5.75 ± 0.34 ^b^	8.52 ± 0.59 ^a^
4	Anethole	1817	MS + RI	—	—	8.74 ± 0.67	7.46 ± 0.83
5	Guaiacol	1860	MS + RI	—	—	6.96 ± 0.44 ^b^	8.02 ± 0.36 ^a^
6	2-Methylnaphthalene	1877	MS + RI	3.11 ± 0.31 ^a^	1.87 ± 0.22 ^b^	2.02 ± 0.18 ^b^	2.93 ± 0.21 ^a^
7	Dimethyl sulfone	1912	MS + RI	0.77 ± 0.09	—	—	1.35 ± 0.14
8	Maltol	1978	MS + RI	—	—	8.51 ± 0.85	8.63 ± 0.67
9	Ethyl maltol	1980	MS + RI	—	—	446.70 ± 45.89 ^b^	453.71 ± 56.77 ^a^
10	Phenol	1992	MS + RI	2.11 ± 0.18 ^a^	1.73 ± 0.16 ^a^	1.59 ± 0.12 ^b^	1.82 ± 0.15 ^a^
11	*p*-Cresol	2089	MS + RI	3.15 ± 0.32 ^c^	3.65 ± 0.40 ^b^	4.10 ± 0.37 ^b^	14.04 ± 1.55 ^a^
12	2,4-Di-tert-butylphenol	2321	MS + RI	1.60 ± 0.20 ^a^	1.03 ± 0.15 ^b^	—	—
13	Indole	2435	MS + RI	—	—	3.47 ± 0.58 ^b^	4.38 ± 0.42 ^a^
	**Subtotal**			12.54 ± 1.3 ^c^	10.05 ± 1.11 ^c^	491.72 ± 49.86 ^b^	520.02 ± 62.61 ^a^
	**Total**			123.85 ± 11.07 ^d^	139.12 ± 10.49 ^c^	739.07 ± 67.68 ^b^	914.01 ± 89.27 ^a^

Note: Each value is expressed as mean ± SD (*n* = 3). Means in the same row with no common superscript differ significantly (*p* < 0.05). The absence of letters indicates no significant difference between groups. LRI: linear retention index on the TR-5MS column. Definitions: MS, mass spectrum (identified with the mass spectra of the database); LRI, linear retention index (compared with the LRI in the online database, http://webbook.nist.gov (accessed on 25 August 2023)). CO (chicken osteopontin), CBES (chicken boneset enzymatic solution), osteopontin reactive cream (ORC), and enzymatic reaction paste (ERP). “—” indicates that the substance has not been detected.

**Table 5:** Effect of different treatment conditions on the aroma components (OAV ≥ 1) of chicken osteopontin.

Compounds	Threshold in Water (ng/g)	OAVs			
		CO	CBES	ORC	ERP
***Aldehyde***					
Octanal	0.41	3	6	—	4
Nonanal	1	20	13	4	8
***Alcohols***					
1-Octen-3-ol	1	—	1	—	—
Linalool	0.01	—	—	554	350
Estragole	6	—	—	2	3
2-Phenylethanol	0.015	61	76	—	—
***Pyrazines***					
3,6-Dimethyl-2-ethylpyrazine	0.4	11	16	6	8
***Pyrrole, thiazole***					
2-Acetylthiazole	3	—	—	2	2
***Sulfurs***					
2-Methyl-3-furanthiol	0.0004	—	—	—	5004
3,3′-Dithiobis-2-methylfuran	0.00002	—	—	—	214,942
***Others***					
2-Methyl-naphthalene	3	1	<1	<1	<1
2-Methoxy-phenol	0.48	—	—	15	17
*p*-Cresol	2.7	1	4	2	5

Note: Each value is expressed as mean ± SD (*n* = 3). The odor thresholds have mainly been obtained from the literature and an online database, with water applied as in the following book: [27]. *Compilations of odor threshold values in air water and other media* (Second enlarged and revised edition). The odor descriptions have mainly been gathered from the following literature and an online database: https://www.flavornet.org (accessed on 25 August 2023). CO (chicken osteopontin), CBES (chicken boneset enzymatic solution), osteopontin reactive cream (ORC), and enzymatic reaction paste (ERP). “—” indicates that the substance has not been detected.

## Data Availability

The original contributions presented in the study are included in the article, further inquiries can be directed to the corresponding author.
